# Effects of Mixed Aerobic, Resistance, and Combined Training on Blood Pressure in Elderly Patients With Hypertension: A Systematic Review and Meta-Analysis of Randomized Controlled Trials

**DOI:** 10.7759/cureus.107875

**Published:** 2026-04-28

**Authors:** Anees A Alyafei, Sameera Alhajri, Ali Q B AlBader, Omar Alsayrafi

**Affiliations:** 1 Preventive Medicine/Wellness Programs, Primary Health Care Corporation, Doha, QAT; 2 Occupational Health and Safety, Primary Health Care Corporation, Doha, QAT; 3 Operations, Primary Health Care Corporation, Doha, QAT; 4 Sports Medicine, Aspetar, Doha, QAT

**Keywords:** aerobic training, combined training, diastolic blood pressure, elderly population, hypertension in the global context, older adult, physical activity, resistance training, systolic blood pressure

## Abstract

Aerobic (AT) and resistance training (RT) are well established as effective strategies for lowering blood pressure (BP) in patients with hypertension; however, evidence on the impact of combined training (CT; AT and RT) on BP remains limited. We therefore conducted a meta-analysis of randomized controlled trials (RCTs) to evaluate the efficacy of AT, RT, and CT for reducing BP in older adults with pre-existing or established hypertension. Five electronic databases (PubMed, CENTRAL, SPORTDiscus, Web of Science, and CINAHL) were systematically searched to identify RCTs reporting the effects of AT, RT, and CT on systolic blood pressure (SBP) and diastolic blood pressure (DBP). RCTs published in English, involving hypertensive older adults and available in peer-reviewed journals as of December 2024, were included. A random-effects model was used for the analysis, with 95% confidence intervals (CIs). Nineteen trials were included, totaling 1,253 participants. SBP was reduced following CT (-4.12 (-6.41 to -1.83; p = 0.0004; I² = 94%), AT (standardized mean difference (SMD) = -1.93 (-5.27 to 1.40); p = 0.06; I² = 96%), and RT (SMD = -2.39 (-2.85 to -1.92); p < 0.00001; I² = 27%). DBP was also reduced following CT (SMD = -4.53 (-6.67 to -2.39); p < 0.0001; I² = 95%), AT (SMD = -0.50 (-3.62 to 2.63); p = 0.04; I² = 8%), and RT (SMD = -0.48 (-1.56 to 0.61); p = 0.05; I² = 39%). Compared with AT and RT as standalone interventions, CT showed a greater BP-reducing effect (SMD = -0.98 (-4.76 to 2.81); p = 0.001; I² = 18%); however, this difference was not statistically robust, likely due to the small number of available trials. Although CT lowered BP in older adults with hypertension, very high between-study heterogeneity (I² > 90% in several analyses) limits confidence in these pooled estimates. The effects of AT on BP outcomes were not significantly different from those of RT.

## Introduction and background

Hypertension, or high blood pressure (BP), is prevalent in older adults and represents a major contributor to cardiovascular diseases (CVDs) [1,2]. It has also been linked to chronic kidney disease, coronary heart disease, stroke, and dementia [3-5]. With the growing aging population, the burden of hypertension has become a significant public health concern. Notably, its prevalence has increased over recent decades, now affecting nearly one-third of the global adult population and approximately 45% of older adults in the United States, according to the American guidelines (systolic blood pressure (SBP)/diastolic blood pressure (DBP) > 130/80 mmHg) [6,7]. According to Mozaffarian et al., an estimated 80 million (33%) US adults are currently diagnosed with hypertension, with the prevalence projected to reach 41.1% by 2030 [7]. Unhealthy diet, obesity, physical inactivity, diabetes, and alcohol use have been identified as risk factors for hypertension [8-10]. However, managing hypertension in older adults remains challenging due to age-related comorbidities, physiological changes, and potential adverse responses to pharmacological treatments. Consequently, nonpharmacological interventions, such as physical exercise (PE), have gained prominence as adjuncts in hypertension management in the elderly population.

Regular PE is widely regarded as an effective lifestyle modification with multiple health benefits, including improved physical function, enhanced cardiovascular health, and better psychological well-being [11]. Among its various forms, aerobic training (AT) and resistance training (RT) have been extensively studied for their effects on hypertension management. AT involves activities that improve the efficiency of oxygen uptake and transport, such as walking, running, and cycling, and is known to reduce both SBP and DBP through mechanisms such as decreased arterial stiffness, improved endothelial function, and increased nitric oxide production [12-14]. In contrast, RT involves exercises that enhance muscular strength and endurance by working against external resistance, such as weightlifting. RT has demonstrated beneficial effects on cardiovascular health, including reductions in BP, through mechanisms such as increased muscle mass, improved vascular function, and enhanced metabolic regulation. It also plays a role in reducing insulin resistance, decreasing inflammation, and improving body composition, all of which contribute to better BP control [15]. Although AT has traditionally been emphasized as first-line exercise therapy for hypertension, emerging evidence suggests that both AT and RT can achieve comparable reductions in BP in many patients [11,16,17].

Despite widespread recommendations for PE in hypertension management, uncertainties remain regarding which exercise modalities, AT, RT, or CT, are most effective for older adults with established hypertension. To date, no meta-analysis has specifically compared AT, RT, and CT using only randomized controlled trials (RCTs) in hypertensive older adults to quantify their relative effects on SBP and DBP. Sabbahi et al. reported that RT and mixed AT may produce similar BP reductions through different physiological mechanisms [10]. Cornelissen and Fagard found that high-intensity PE significantly lowers BP [18], although excessively intense exercise may lead to adverse effects [19]. Recent evidence suggests that high-intensity interval training (HIIT), compared with moderate-intensity training (MIT), may significantly reduce DBP and improve maximal oxygen uptake in hypertensive adults. However, other studies indicate that while high-volume HIIT may improve weight loss, it does not consistently result in significant BP reductions across different training volumes [13,14]. Furthermore, although strong evidence supports the BP-lowering effects of AT, findings regarding RT as a standalone therapy remain more variable [20]. Recent studies also suggest that CT may offer synergistic benefits, potentially leading to greater reductions in BP than either modality alone. This may be explained by their complementary effects: AT enhances cardiovascular fitness, while RT improves muscular strength and overall physical function.

Despite the growing body of evidence, a rigorous systematic review and meta-analysis (SRMA) is needed to synthesize data from RCTs and provide clearer conclusions on the effectiveness of AT, RT, and CT in adults with hypertension. RCTs are considered the gold standard for evaluating interventions due to their ability to minimize bias and support causal inference. Accordingly, the primary objective of this review is to assess the efficacy of AT, RT, and CT in reducing BP in older adults with hypertension. Specifically, this study aims to quantify the effects of these three exercise modalities on BP outcomes and to clarify their respective roles in hypertension management in this population.

## Review

Methods

This meta-analysis was conducted in accordance with the 2020 Preferred Reporting Items for Systematic Reviews and Meta-Analyses (PRISMA) guidelines, ensuring methodological rigor, transparency, and reproducibility throughout all stages of the review process [21]. The protocol was prospectively registered in the International Prospective Register of Systematic Reviews (PROSPERO) under registration number CRD420250649985. Supplementary material 1 provides additional methodological details.

Data Source and Search Strategy

PubMed (MEDLINE), CINAHL, the Cochrane Library (CENTRAL), SPORTDiscus, and Web of Science were comprehensively searched. Each database was searched from its inception; however, inclusion was restricted to studies published between 2015 and 2025. Only studies published in English were considered, and all relevant studies available through December 2024 were screened for possible inclusion. In consultation with a medical librarian, a search strategy based on a predefined participants, intervention/exposure, comparison, and outcome (PICO) framework was developed to ensure a thorough and systematic approach [22] (Table 1).

**Table 1:** PICO framework

PICO framework	
Participants (P)	Hypertensive aging/elderly adults, i.e., diagnosed previously or continuing treatment for hypertension; mean age 64.4 ± 9.8 years (range 55-90)
Intervention (I)	Aerobic training (AT), resistance/strength training (RT), and combined training (CT)
Comparison (C)	(1) Exercise modalities (aerobic vs. resistance, aerobic vs. combined, resistance vs. combined); (2) non-exercise intervention, control group (CG) (aerobic vs. control group, resistance vs. control group, combined vs. control group)
Outcome (O)	Blood pressure (BP), systolic (SBP), and diastolic (DBP) changes

The keywords and medical subject headings (MeSH) used in the database search included “high blood pressure”, “hypertension”, “older adults”, “elderly”, “physical exercise”, “exercise”, “aerobic exercise”, “mixed aerobic exercise”, “resistance training”, “strength training”, “combined exercise”, “randomized controlled trial”, “RCT”, and “meta-analysis”. Boolean operators (OR and AND) were used to combine terms and refine each database search. We also reviewed the reference lists of relevant reviews to identify additional articles. The search strategy was reviewed by a specialist in systematic review methodology (Supplementary material 2).

Study Eligibility

RCTs were eligible for inclusion if they were published in English, available in full text, and published between 2015 and 2025. Eligible studies included hypertensive older adults (male and female), with a mean age of 64.4 ± 9.8 years (range: 55-90 years). Studies were required to involve participants in supervised, structured exercise interventions, including RT, AT, or CT, and to report BP, SBP, and DBP as primary outcomes, with measurements obtained before and after the intervention. No restrictions were placed on session duration, intervention length, modality, or weekly frequency and volume. Eligible trials were required to include at least one comparison group, either a control (non-exercise) group or an alternative exercise modality (e.g., CT). Studies were excluded if they lacked a comparison group, reported only pre- and post-intervention outcomes without between-group comparisons, or investigated other primary cardiovascular diseases. Additional exclusion criteria included unpublished studies, studies published before 2015, conference abstracts or protocols without full data, and studies published in languages other than English. Comorbidities such as diabetes, obesity, or overweight status were not considered exclusion criteria.

Study Selection and Data Extraction

Two reviewers independently screened the titles and abstracts of all records identified through the search to assess potential eligibility. When uncertainty arose regarding inclusion, the full text of the article was retrieved for further evaluation. Full-text articles of all potentially eligible studies were stored electronically in Mendeley and reviewed independently according to the predefined inclusion and exclusion criteria. Only studies published in English were considered. Any discrepancies between reviewers were resolved through discussion and consensus or, when necessary, through consultation with a senior member of the research team.

Following the initial screening, the same two reviewers independently assessed the full texts of all potentially relevant studies to confirm eligibility. Disagreements were resolved through discussion or, if consensus could not be reached, by consultation with a third reviewer. Data on study characteristics, participant demographics, intervention components, and outcomes were extracted using a standardized data collection form.

Risk-of-Bias Assessment and Quality of Evidence

The risk of bias in RCTs was assessed using the Cochrane Risk of Bias Tool, which evaluates methodological quality across six domains: selection, performance, detection, attrition, reporting, and other sources of bias [23]. Each study was classified as having a low, unclear, or high risk of bias within these domains. The overall certainty of the evidence was subsequently assessed using the GRADE approach [24]. This framework evaluates the quality of evidence for each outcome based on risk of bias, directness, consistency of findings, precision of effect estimates, and the likelihood of publication bias.

Statistical analysis

Data and statistical analyses were performed using the Cochrane Collaboration’s Review Manager (RevMan 5.4; The Cochrane Collaboration, London, England, UK) [25]. For continuous outcomes measured on the same scale across studies, pooled effects were calculated as standardized mean differences (SMDs) with 95% confidence intervals (CIs). Cochran’s Q test and the associated p-value were used to assess heterogeneity and overall effects on BP outcomes in each meta-analysis. Statistical significance was defined as a two-sided p-value ≤ 0.05, and heterogeneity was considered substantial when I² ≥ 50%. When substantial heterogeneity was identified, subgroup and sensitivity analyses were conducted to explore potential sources of variation [26,27].

Sensitivity Analyses

Sensitivity analyses were performed to evaluate the influence of study methodological quality by excluding studies judged to be at high risk of bias and by comparing results obtained using fixed-effect and random-effects models. These analyses were conducted using the Cochrane Review Manager (RevMan) platform. Forest plots were generated to display between-group differences and corresponding 95% CIs for resting SBP and DBP.

Publication Bias

Potential publication bias is reported in the Results section. The risk of publication bias was assessed by visually examining funnel plots for asymmetry. The degree of asymmetry was further evaluated using Egger’s test [28]. Statistical significance was defined as a p-value < 0.05.

Results

Search Strategy Results

**Figure 1:**
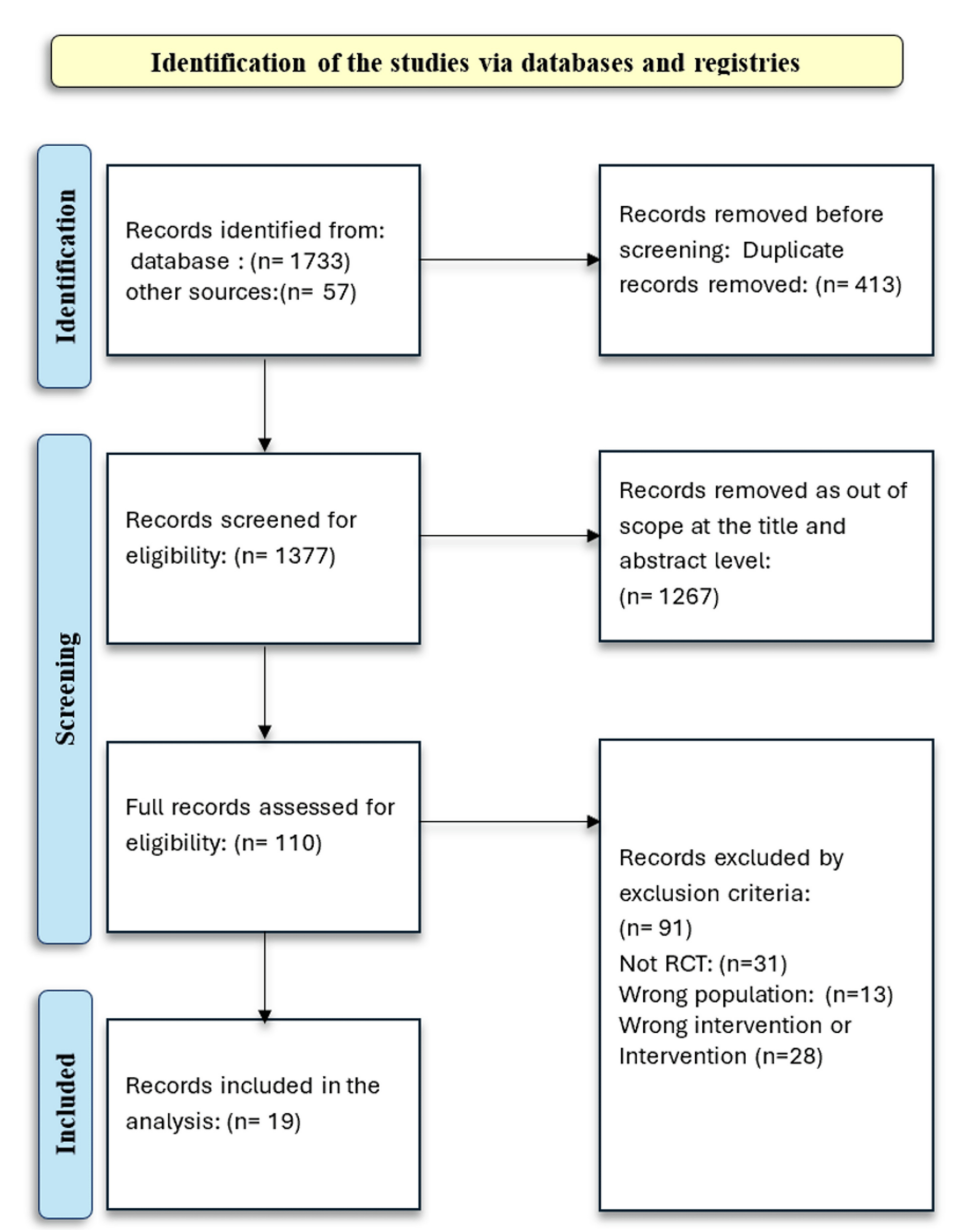
PRISMA flow diagram PRISMA: Preferred Reporting Items for Systematic Reviews and Meta-Analyses, RCTs: randomized controlled trials.

The PRISMA flow diagram for study identification and selection is presented in Figure 1. The initial database search yielded 1,790 records, of which 413 duplicate and clearly ineligible entries were removed. After title and abstract screening, an additional 1,267 articles were excluded as out of scope. Of the 110 articles retrieved for full-text review, 91 were excluded because they met the exclusion criteria. Finally, 19 studies involving adults with a mean age of approximately 60 years and diagnosed with hypertension or receiving treatment for hypertension were included in this analysis.

Study and Intervention Characteristics

Across all included studies, 1,253 participants were analyzed, including 389 in mixed AT groups, 300 in RT groups, 132 in CT groups, and the remainder in control or other comparison groups. Most studies included older adults of both sexes, with only one study enrolling exclusively male participants [29]. The pooled mean age of participants across studies was 64.4 ± 9.8 years, and most individual trials included adults aged 50-80 years. Five studies involved participants receiving antihypertensive medications. Only 11 studies explicitly reported criteria for defining clinical hypertension or high BP.

Regarding intervention characteristics, most studies reported a follow-up duration of 12 weeks, with varying training frequencies and intervals. Most exercise sessions lasted 10-60 minutes and were conducted 3-4 times per week. AT interventions included activities such as exergaming, walking, cycling, treadmill running, and hula dancing. RT interventions included exercises such as chest press, knee and elbow flexion, squats, bench press, and deadlifts, among others. Although some trials reported antihypertensive medication use, the available data were insufficiently detailed and inconsistent to allow reliable subgroup analyses comparing medicated and non-medicated participants. General characteristics of the included studies and exercise interventions are summarized in Table 2.

**Table 2:** Summary of key study characteristics and intervention features

Author, publication year (Country)	Study design	Participant characteristics		PE interventions				Diagnostic criteria for hypertension	Antihypertensive treatment	Outcome	Conclusions
		Sample (N), sex (M/F), age (mean years ± SD)	Population type	Modality	Period	Session duration and frequency	Controls (non-exercise group)				
Bavaresco Gambassi et al. (2024) [30] (Brazil)	Interventional, controlled, RCT	N = 26 (CT: 13; CG: 13) 68.4 ± 8.3 years	Older adults diagnosed with grade 1 hypertension (HTN) not engaged in regular exercise training for the past 6 months	Vertical chest press, plantar flexion, seated row, hip abduction, elbow flexion, trunk flexion, and chair squat	8 weeks with two sessions per week	CT was conducted twice weekly for 8 weeks, with at least 48 hours of rest between sessions within the same week	No exercise program for 8 weeks	Not reported	None	Pulse pressure (PP), central pulse pressure, pulse wave velocity 4. SBP, DBP, central SBP, central DBP	Explosive resistance exercise using elastic bands, when paired with endurance training, lessens arterial stiffness and produces marked improvements in key hemodynamic indices
Hou et al. (2023) [31] (China)	Randomized, assessor-blinded, three-armed clinical trial	N = 128 (AT/exergame group: 41, RT/bicycle exercise: 44, CG: 43)	Older patients with a history of hypertension	AT/exergame: Nintendo, Wii Sports games, followed by tennis, bowling, baseball, and golf. RT consisted of sitting on a recumbent bicycle and pedaling at a comfortable cadence, using the first gear for women and the second gear for men	The intervention groups trained for 60 minutes per class, three times weekly, over a 16-week period	The intervention comprised a 16-week supervised program with three 1-hour sessions per week, each session structured as a 5-minute warm‑up, 50 minutes of training, and a 5-minute cool-down	Maintained their normal lifestyle, such as a normal diet and natural activity, with no new form of exercises	Clinical hypertension was defined as a systolic blood pressure (SBP) of ≥140 mmHg and/or a diastolic blood pressure (DBP) of ≥90 mm Hg, with confirmation on three separate clinic visits conducted on different days	Yes	Blood pressure (SBP, DBP), working memory	Compared with the control group, both intervention groups achieved significant gains in working memory; however, the extent of blood pressure reduction did not differ significantly between them
Bavaresco Gambassi et al. 2023 [32] (Brazil)	Crossover RCT	N = 18 (CT: 9; CG: 9) ≥60 years	Older adults clinically diagnosed with hypertension	The CT protocol comprised vertical chest press, seated row, hip abduction, plantar flexion, and trunk flexion exercises, all performed using Thera-Band professional latex resistance bands	N/A	The DERE protocol comprised five pairs of consecutive exercises performed back-to-back, with no complete rest intervals during the entire session	No exercise was performed; participants were instructed to lie still for 25 minutes	Not reported	None	PP, SBP, and DBP	There was a significant decrease in PP and SBP. Hence, RT improved SBP in hypertensive older adults
Abrahin et al. (2024) [33]	Single-blinded RCT	N = 68 (RT: 27; CG: 25) ≥60 years	Older adult participants presenting hypertension stage 1 or 2	The RT protocol comprised two sets of 6-10 repetitions at approximately 70%-85% of 1 RM, incorporating bench press (machine), deadlift (barbell), rowing (machine), standing calf raises (machine), leg curls (machine), and abdominal reverse crunches	12 weeks	The RT group underwent a 12-week training phase (weeks 3-14), followed by functional reassessment and blood sampling in weeks 15-16, with sessions lasting 30-40 minutes on three nonconsecutive days per week, each session comprising two sets of 6-10 repetitions	Participants were instructed to refrain from engaging in exercise for 16 weeks	Not reported	Yes, antihypertensive therapy included angiotensin receptor blockers, β-adrenergic blockers, diuretics, and calcium channel blockers	Outcomes assessed were blood pressure, functional performance (timed up and go, handgrip strength, biceps curl, and sit-to-stand), fasting glucose, and lipid profile parameters	Resistance training enhanced functional performance in older adults with hypertension and effectively lowered systolic blood pressure, although it did not produce measurable changes in biochemical outcomes
Abrahin et al. (2022) [34]	Crossover RCT	N = 61 (RT: 20; AT: 20; CG: 21) ≥60 years	Older patients presenting with hypertension stage 1 or 2	The RT protocol included resistance exercises such as barbell deadlifts, unilateral machine rowing, standing calf raises, leg curls, and abdominal reverse crunches, whereas AT consisted of ergometer cycling performed at varying intensities	12 weeks	The RT protocol involved 30-40-minute resistance training sessions performed three nonconsecutive days per week, whereas AT was also performed on three nonconsecutive days per week over a 12-week period	No exercise intervention	Not reported	Yes; participants were treated with antihypertensive medications, including angiotensin receptor blockers, adrenergic blockers, diuretics, and calcium channel blockers	Blood pressure, functional performance, glycated hemoglobin, lipid profiles	Responses to RT or AT varied among older adults with hypertension; however, both RT and AT effectively reduced SBP
Gargallo et al. (2022) [35] (Spain)	Crossover RCT	N =19; RT (mild: 19, moderate: 19, intense: 19; CG: 19); 64.53 (55-70) years	Older patients with hypertension	Resistance training: three (mild), six (moderate), and nine high-intensity sets of 20-repetition maximum (RM) of a single-elbow flexion exercise with bands	N/A	All participants completed five sessions: one familiarization session to determine exercise intensity and four experimental resistance training sessions involving a biceps curl (elbow flexion) exercise, each performed on a separate day with a different training volume (one condition per session)	No exercise	Hypertension was defined as SBP ≥ 130 mmHg or DBP ≥ 80 mmHg	None	SBP, DBP, heart rate (HR)	Performing six and nine sets of 20 RM elicited statistically significant reductions in SBP at 30- and 60-minute post-exercise measurements
Sardeli et al. 2022 [36] (Brazil)	Prospective interventional RCT, with a parallel control group	N = 52 (CT: 23; CG: 23) 65.4 ± 4.3 years	Hypertensive older adults	AT consisted of 50 minutes of continuous walking and/or running on a treadmill, whereas ST comprised a single set of 15 repetitions for each exercise (leg extension and flexion, leg press, heel raise, bench press, pulley, and abdominal exercises)	16 weeks	Twice a week, strength training (15 min) was followed by AT (50 min)	Individuals did not participate in any treatment	Not reported	Yes (angiotensin receptor blockers, β-adrenergic blockers, diuretics, and calcium channel blockers)	Outcomes assessed included BP (SBP and DBP), physical fitness, and a range of hemodynamic, autonomic, inflammatory, oxidative, glucose, and lipid mediators	Although the training program improved cardiorespiratory fitness and muscular strength, it did not lead to reductions in blood pressure or in the broader panel of mediators among hypertensive older adults
Caminiti et al. (2021) [20]	Two-arm RCT	N = 55 M (AT: 27; CT: 28) 69 ± 10.4 years	Hypertensive patients enrolled in a cardiac rehabilitation program	AT sessions consisted of a 10-minute warm‑up, 60 minutes of treadmill‑based aerobic exercise, and a 10-minute cool-down, whereas CT sessions involved 40 minutes of treadmill aerobic training followed by 20 minutes of resistance exercises (leg press, leg extension, shoulder press, chest press, low row, and vertical traction)	12 weeks	Training was three sessions per week, each lasting 80 minutes	A non-exercise control group was not included	Hypertension (BP > 140/90 mmHg)	None	Short-term BP variability	CT was more effective than AT in lowering short-term BPV, suggesting that CT may be the more suitable exercise modality when the goal is to reduce BPV in addition to BP levels
Kaholokula et al. (2021) [37] (Hawaii)	Two-arm RCT	N = 263 (AT: 131; CG: 132); 58.1 ± 13.7 years	Native Hawaiians with uncontrolled hypertension (HTN)	AT (hula dance)	6 months	The intervention group completed a 6‑month Ola Hou i ka Hula program delivered in two phases, with the initial 3 months consisting of 1-hour dance sessions held twice per week (24 sessions total)	Participated in a 1-week education class during the 6-month intervention	Systolic BP ≥ 140 mmHg (or ≥ 130 mmHg	None	BP (systolic BP and diastolic BP in mmHg)	The intervention yielded greater reductions in systolic and diastolic BP; 43% of the AT group and 21% of the control group achieved a BP < 130/80 mmHg (p < 0.001)
Lopes et al. (2021) [38] (Portugal)	A two-arm, prospective, two-center, single-blinded RCT	N = 53 (AT: 26; CG: 27)	Adults aged 40-75 years diagnosed with resistant hypertension	AT consisted of cycling and/or walking at 50%-70% of VO₂ max (corresponding to a rating of 11-14 on the Borg scale), followed by a 10-minute cool-down	12 weeks	The AT program consisted of three sessions per week, with each session including a 10-minute warm-up, 40 minutes of cycling and/or walking, and a 10-minute cool-down	No exercise	A mean 24-hour ambulatory systolic BP of 130 mmHg or greater and/or a mean daytime systolic BP of 135 mmHg or greater	Yes	24-hour ambulatory systolic BP change, heart rate, body composition, cardiorespiratory fitness, and adverse events	The 12-week aerobic exercise program produced reductions in 24‑hour and daytime ambulatory BP, as well as in office systolic BP, among patients with resistant hypertension
Iellamo et al. (2021) [39] (Italy)	RCT	N = 36 (AT: 12; RT: 12; CT: 12) 66 ± 4.7 years	Elderly sedentary male patients with hypertension (lasting more than 1 year)	Aerobic continuous exercise (AT), high-intensity interval exercise (RT), and combined (aerobic and resistance) exercise (CT)	12 weeks	Each exercise session in the training program consisted of a 10-minute warming-up, 40 minutes of treadmill-based AT at 55%-70% of VO₂ max, and a 10-minute cool-down	No exercise	Not specified	None	24-hour and nocturnal systolic and diastolic blood pressure (BP), post-exercise hypotension (PEH)	AT and CT led to greater PEH than RT did, but intensive RT led to greater and more sustained PEH
Da Silva et al. (2021) [40]	Crossover RCT	N = 11 (RT: 11 CG: 11) 65.8 ± 4.6 years; 7 M/4 F	Older individuals with a diagnosis of hypertension or whose BP is controlled with medication	RT consisted of six exercises: sit-to-stand, bar-supported push-up, step-ups, elastic-tube row, single-leg hip raise, and abdominal exercise	A total duration of 43 minutes, consisting of a 5-minute warm-up followed by 38 minutes of ST	The training session consisted of six exercises (three sets, 30 seconds per exercise)	No expressive movements were performed, and participants remained at rest for the same duration as the training session	Not reported	None	Systolic BP (SBP) and diastolic BP (DBP)	Compared with the control session, the RT session results in lower SBP at 30 minutes post-PE and is associated with a positive affective response in hypertensive older adults
Schimitt et al. (2020) [41] (Brazil)	RCT with crossover design	N = 24 (RT: 23 CG: 23) 66.7 ± 4.3 (60-75) years	Older adults with essential hypertension	The RT protocol consisted of 3 sets of 8-10 repetitions for each of five exercises: leg press, bench press, knee extension, upright row, and knee flexion		Session began between 2 and 3 PM, lasting for 2 hours; with each session having 20 mins of rest, and an additional 40 mins of RT or control protocol, and 60 mins of rest after	Participants sat on the same exercise machine but did not do any exercise	Not reported	None	Difference in 24 h ambulatory BP, hemodynamic response	A single bout of RT lowered office BP, but this post-exercise hypotensive effect did not persist under ambulatory monitoring conditions in older adults with essential hypertension
Sócrates et al. 2020 [42] (Brazil)	Single-blind, two-arm, parallel-group controlled RCT	N = 40 (AT: 20; CG: 20) 64.4 ± 3.6 years	Older hypertensive women on medication treatment	Participants first completed a maximal graded exercise test on a motorized treadmill, followed by an incremental ramp protocol beginning at 2.5 km/h with a 4% grade and progressing continuously to 5.5 km/h and a 14% grade by 10 minutes	8 weeks	AT sessions were conducted three times per week, lasting 30-50 minutes each (n = 20)	Participants also attended group-based health education sessions once per week throughout the 8-week intervention period	Not specified	None	Ambulatory BP; six-minute walking test performance	AT, i.e., self-selected training intensity (SSTI) is effective at inducing an active lifestyle and improving fitness in hypertensive adults, but does not improve BP control in the short-term
Schroeder et al. (2019) [43] (United States)	RCT	N = 69 (CT: 18; AT: 17; RT: 17; CG: 17) 58 ± 7 years	Adults with elevated blood pressure or hypertension who were overweight or obese and were mainly sedentary	AT consisted of treadmill or cycle ergometer exercise; RT included chest/shoulder press, pulldown, lower-back extension, biceps curl, and leg press; CT combined 30 minutes of aerobic exercise with 30 minutes of resistance training per session	8 weeks	The 8-week program was delivered 3 days per week, with either 60-minute aerobic sessions, 60-minute resistance sessions, or combined sessions consisting of 30 minutes of aerobic exercise followed by 30 minutes of resistance training	No exercise activity	Blood pressure was in the systolic/diastolic range of 120-149/80-99 mmHg in the absence of antihypertensive medication	None	Outcomes assessed included peripheral and central BP, cardiorespiratory fitness (CRF), muscular strength, body composition, blood glucose, and lipid profile measures	Combined training may offer more comprehensive cardiovascular benefits than time-matched AT or RT alone
de Oliveira et al. [44] 2020 (Brazil)	Single-blinded RCT	N = 23 (CT: 13; CG: 10) 62.65 ± 6.4 years	Elderly individuals with HT	AT consisted of treadmill walking, whereas RT involved upper-limb exercises (rowing, standing bench press, arm curl) and lower-limb exercises (knee extension, knee flexion, and forward walking using a resistance band)	8 weeks	AT consisted of 25 minutes of treadmill exercise, with intensity increased by 5% every two weeks, whereas RT comprised 2 sets of 15 repetitions with 30-second rest intervals between sets and exercises	No exercise	Hypertension (<130/80 mmHg)	None	Outcomes included resting BP, peak oxygen consumption (VO_2_ peak), and right knee and elbow flexion strength	RT lowered BP and enhanced both cardiorespiratory fitness and upper-limb muscular strength in hypertensive older adults
Ruangthai et al. (2019) [45] (Thailand)	Single-blinded RCT	N = 54 (CT: 16; AT: 13; RT: 13; CG: 12) 67 ± 5.8 years (12 M/55 F	Elderly individuals with HT	Participants in the AT group performed walking with arm raises, heel kicks, tiptoe walking, arm adduction/abduction, and knee lifts; those in the RT group completed squats, leg raises, knee extension and flexion, hip adduction, leg kickbacks, shoulder press, bench press, biceps curls, triceps dips, sit-ups, and back extensions; and those in the CT group undertook both the AT and RT exercise routines	The intervention lasted 12 weeks and comprised two phases: an initial 12-week supervised program with three sessions per week, followed by a self-supervised phase during which participants exercised independently while receiving weekly phone calls for 12 weeks to support and reinforce regular exercise	AT: 3 times weekly for 60 min per session with 40-min walk (50%-60% intensity) in weeks 1-6, and 60%-70% intensity after 6 weeks. RT: 3 times weekly for 60 min per session, with loads of 50%-70% and 15 repetitions per set for 3 sets in the first 6 weeks, and 60%-80% and 10 repetitions per set for 3 sets from week 7	No exercise	Hypertension was defined as SBP ≥ 130 mmHg or DBP ≥ 80 mmHg	None	Blood pressure; body composition; glutathione peroxidase; total nitrite/nitrate, lipid peroxidation, blood glucose, and lipid profiles	Following the self-supervised training phase, CT yielded superior outcomes, likely driven by higher exercise adherence and session attendance among elderly individuals with hypertension
Chan et al. (2018) [46] (China)	3-arm RCT	N = 264 (AT (brisk walking):82 RT (Tai Chi: 82; CG: 82) 64.4 ± 9.8 (30-90) years	Adults with hypertension and at least two but not more than three modifiable cardiovascular disease risk factors (diabetes, dyslipidemia, overweight, physical inactivity, and smoking)	The Tai Chi group attended a 24-form Yang Style Tai Chi class for 60 minutes, 2 times a week for 3 months. The risk walking group was instructed to walk between 5 and 6 km/h for 30 minutes a day on at least 5 days per week	3 months (12 weeks)	The Tai Chi and brisk walking groups engaged in moderate-intensity physical activity 150 min/week for 3 months; daily home-based practice was encouraged for another 6 months	No Additional physical activity was recommended	Not specified	None	Blood pressure, high- and low-density lipoprotein, quality of life (psychological well-being)	Tai Chi was superior to brisk walking in reducing several cardiovascular disease risk factors and enhancing psychosocial well-being and can therefore be recommended as a viable exercise to support a healthy lifestyle
Dantas et al. (2016) [47] Brazil)	RCT	N = 25; (RT: 13; CG: 12), mean age = 66.1 (60-75) years	Non-smoking, insufficiently active hypertensive elderly women	The RT program incorporated seated leg press, seated rowing, trunk flexion, knee flexion on a machine, bench press, trunk extension on a machine, push press, standing plantar flexion, and front pulldown exercises	10 weeks	Sessions were held twice a week for the first five weeks, then three times a week thereafter	No exercise activity	Not reported	None	Outcomes included mean blood pressure (MBP), plasma malondialdehyde (MDA), total antioxidant capacity (TAC), plasma nitrite (NO₂⁻), and forearm blood flow (FBF)	The strength exercises reduced oxidative stress in hypertensive older adults females and this reduction was moderately associated with additional cardiovascular benefits

Quality Assessment and Risk of Bias

The certainty of evidence for all included RCTs was appraised using the GRADE framework, which considers risk of bias, inconsistency, indirectness, imprecision, and publication bias. Overall, the evidence base was rated as high quality, with only three trials downgraded to moderate quality due to limitations such as unclear blinding procedures and incomplete reporting of intervention characteristics. Studies rated as providing moderate-quality evidence lacked precise intervention duration data and had an unclear risk of bias related to participant and personnel blinding [32,35,40] (Supplementary material 3). All included studies reported appropriate methods for random sequence generation and allocation concealment and, overall, demonstrated a low risk of bias across these domains. However, in most studies, the risk of bias related to blinding of participants and personnel was judged as unclear.

**Figure 2:**
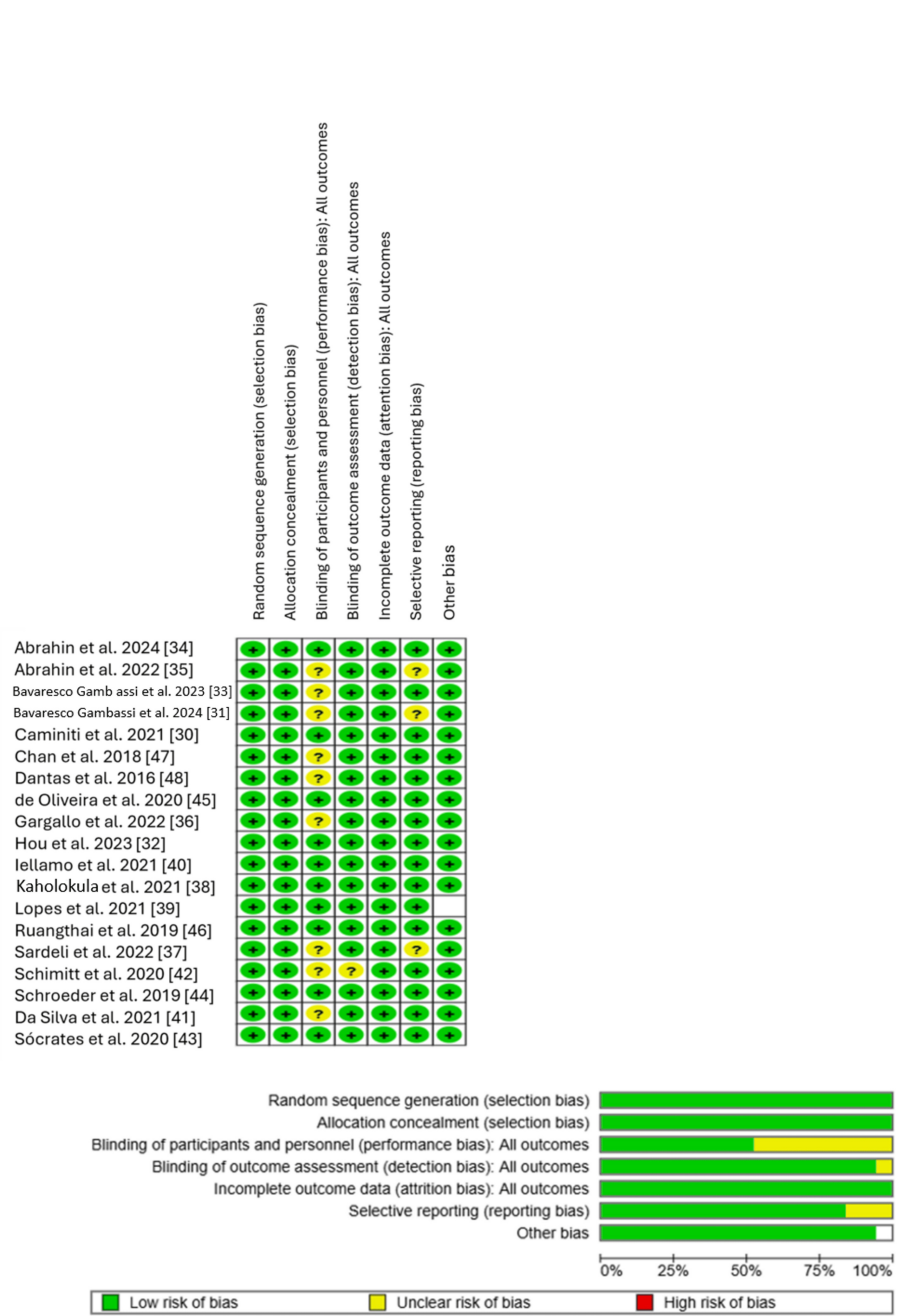
Summary of risk-of-bias assessment Most domains showed predominantly low risk of bias across the included RCTs, particularly for random sequence generation, allocation concealment, and attrition and reporting biases. However, blinding of participants and personnel (performance bias) was frequently unclear, indicating some potential vulnerability to bias in subjective or behaviorally influenced outcomes.

A summary of the overall risk of bias for all included RCTs is provided in Figure 2.

Quantitative Synthesis: Effects of Exercise Interventions on BP Outcomes

**Figure 3:**
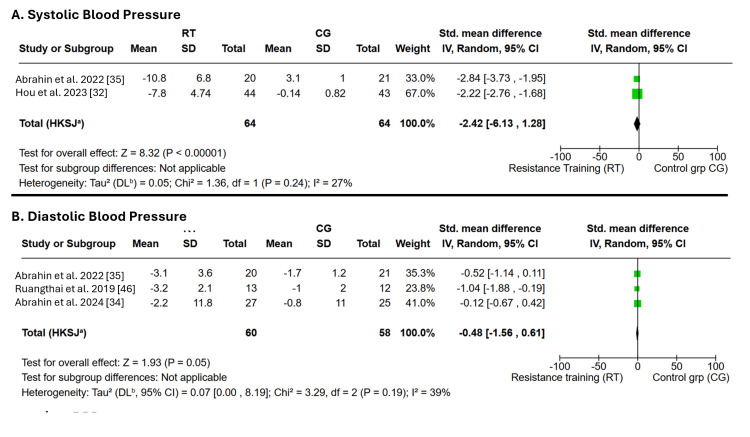
Forest plot showing the effects of resistance training on blood pressure RT: resistance training, CG: control group, SBP: systolic blood pressure, DBP: diastolic blood pressure, IV: inverse variance, HKSJ: Hartung-Knapp-Sidik-Jonkman, DL: DerSimonian-Laird.

Effects of RT alone on BP: Six studies evaluated the effects of RT on ambulatory BP [31,33,34,43,45,47]. The meta-analysis suggested a reduction in both SBP and DBP; however, the pooled effects were not statistically significant (p > 0.05), and very high heterogeneity (I² > 90%) indicated substantial between-study variability. To explore this variability, leave-one-out sensitivity analyses were performed. However, substantial heterogeneity persisted and could not be fully explained. Because the limited number and variability of covariates across individual studies precluded meaningful subgroup analyses, leave-one-out sensitivity analysis was used to assess the influence of sequential exclusion of individual trials on the pooled estimates and to better understand the observed heterogeneity. RT was associated with a significantly greater reduction in SBP compared with the control or non-intervention condition (Z = 10.14; p < 0.00001), whereas its effect on DBP was not statistically significant (Z = 1.93; P = 0.05). Low heterogeneity was observed in the SBP (I² = 27%) and DBP (I² = 39%) analyses, suggesting consistency across the included studies. The robustness of the findings is supported by the SMD and 95% CIs, with effects of SMD = -2.39 (-2.85 to −1.92) and SMD = -0.48 (-1.56 to 0.61), respectively, as shown in Figure 3.

**Figure 4:**
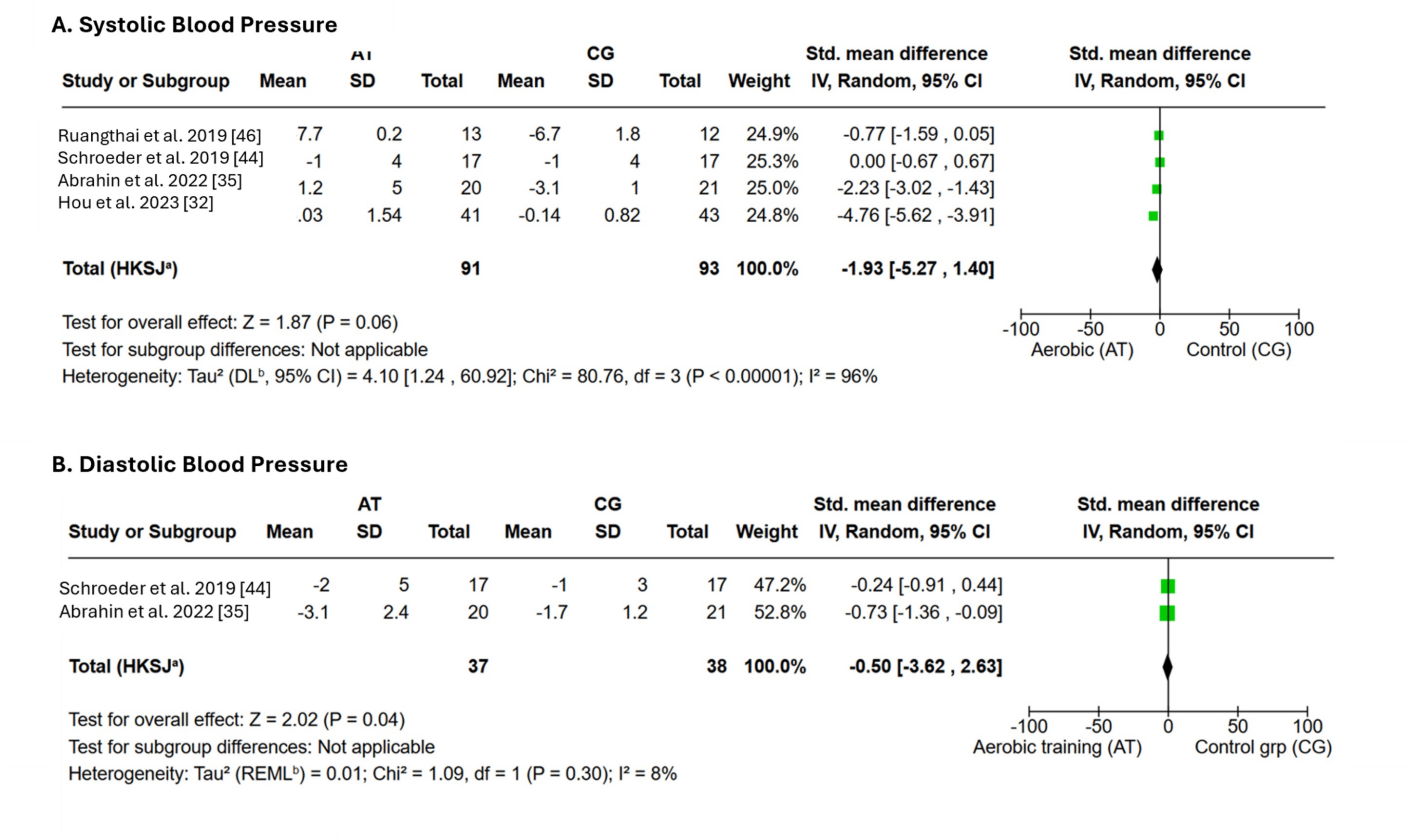
Forest plot showing the effects of aerobic training on blood pressure AT: aerobic training, CG: control group, SBP: systolic blood pressure, DBP: diastolic blood pressure, IV: inverse variance, HKSJ: Hartung-Knapp-Sidik-Jonkman, DL: DerSimonian-Laird, REML: restricted maximum likelihood.

Effects of AT alone on BP: Overall, AT reduced both SBP and DBP, as reported in four studies involving 184 participants [31,34,43,45]. For SBP, the pooled analysis suggested a reduction (Z = 1.87, p = 0.06), which did not reach statistical significance (SMD = -1.93, 95% CI -5.27 to 1.40). However, substantial heterogeneity was observed (I² = 96%), indicating considerable variability among the included studies. The meta-analysis found no statistically significant effect of AT on DBP, with high heterogeneity suggesting limited generalizability of the results. Sensitivity analysis, excluding two studies, confirmed a nonsignificant effect of AT on DBP in hypertensive patients (SMD = -0.50, 95% CI -3.62 to 2.63, Z = 2.02, p = 0.04). As depicted in Figure 4, statistical heterogeneity across the included studies was minimal in this sensitivity analysis (I² = 8%), indicating no meaningful between-study inconsistency.

**Figure 5:**
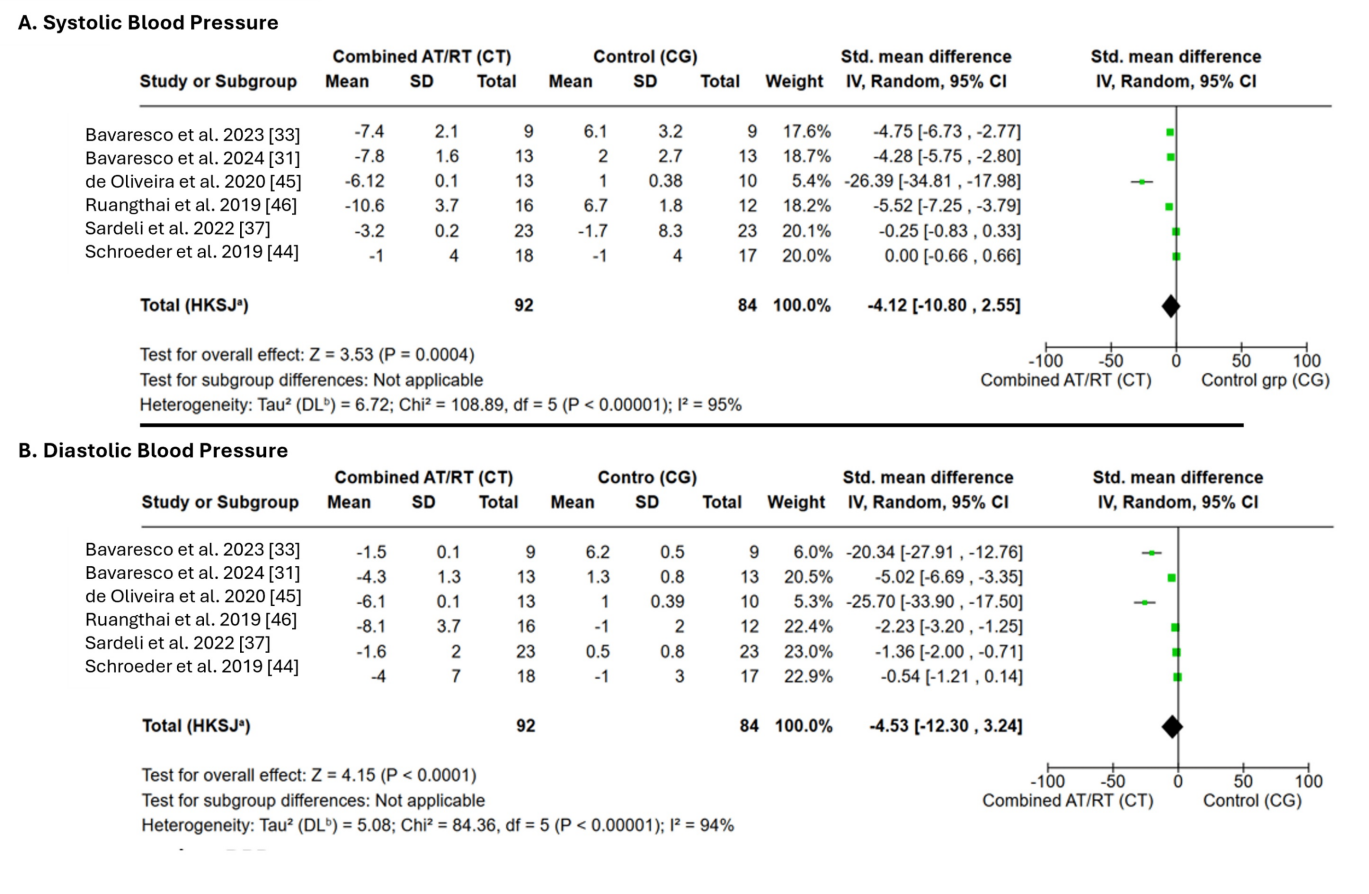
Forest plot showing the effects of combined training on blood pressure vs. no exercise AT: aerobic training, RT: resistance training, CT: combined training, CG: control group, SBP: systolic blood pressure, DBP: diastolic blood pressure, IV: inverse variance, HKSJ: Hartung-Knapp-Sidik-Jonkman, DL: DerSimonian-Laird.

Effects of CT on BP vs. no exercise: Six RCTs, including a total of 176 participants, investigated the effects of CT on BP [30,32,36,43-45]. The pooled meta-analysis results showed that, compared with no exercise, CT significantly reduced both SBP and DBP. The overall effect size, expressed as SMD, was -4.12 (95% CI -6.41 to -1.83; p = 0.0004) for SBP and -4.53 (95% CI -6.67 to -2.39; p < 0.0001) for DBP. Both effects were statistically significant (p < 0.001). However, very high heterogeneity (I² = 95%) indicated substantial interstudy variability, which limits the generalizability of these pooled estimates and suggests that the findings should be interpreted with caution (Figure 5).

**Figure 6:**
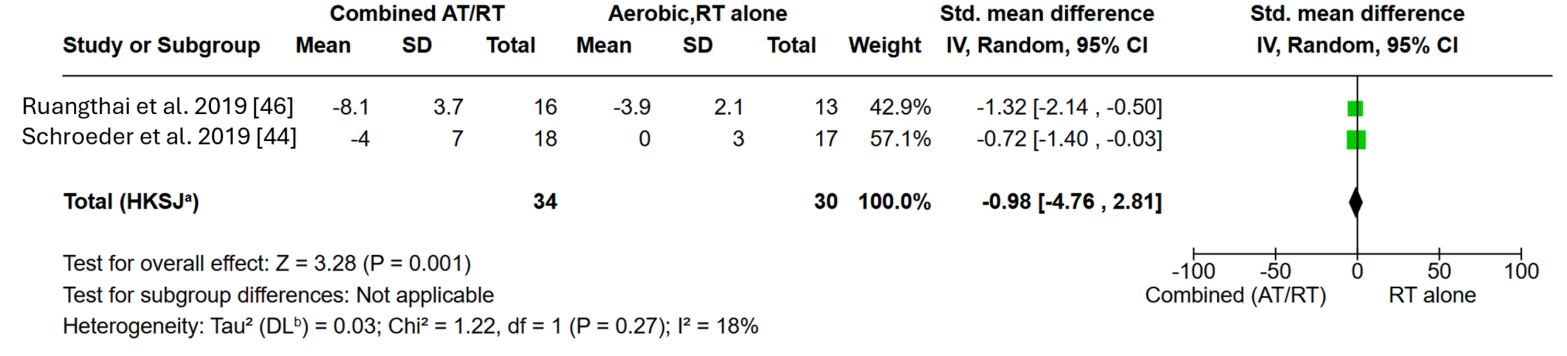
Forest plot showing the effects of combined training vs. resistance training on blood pressure AT: aerobic training, RT: resistance training, IV: inverse variance, HKSJ: Hartung-Knapp-Sidik-Jonkman, DL: Der Simonian-Laird.

Effects of CT on BP vs. AT or RT: In this study, the effects of CT (aerobic plus resistance) on SBP and DBP were compared with AT or RT alone. Among the 21 included trials, seven compared CT with either AT or RT and generally reported improvements in both SBP and DBP with CT; however, these between-group differences were not statistically significant, and substantial heterogeneity precluded a pooled meta-analysis. A separate meta-analysis including two studies (64 participants) compared CT with RT alone and demonstrated that CT produced a significantly greater reduction in SBP, with an SMD of -0.98 (95% CI -1.40 to -0.56; Z = 3.28; p = 0.001) and low heterogeneity (I² = 18%), indicating consistent effects across studies (Figure 6).

Risk of Bias Across Studies/Publication Bias

**Figure 7:**
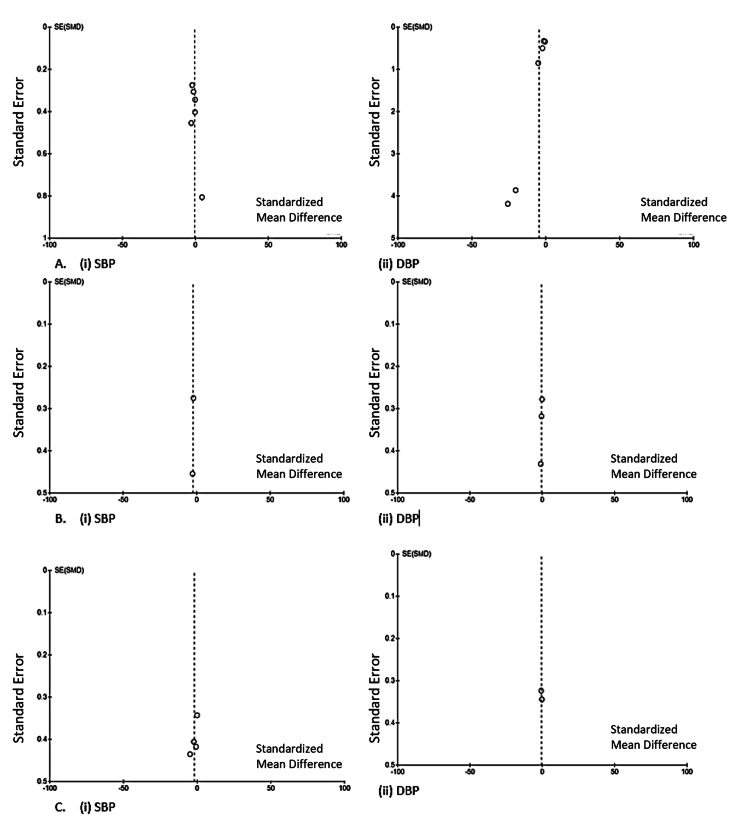
Egger's funnel plot analysis SBP: systolic blood pressure, DBP: diastolic blood pressure.

Visual assessment indicated low attrition bias and no evidence of publication bias. Egger’s funnel plots were used to assess publication bias for each outcome (Figure 7).

Discussion

Summary of Findings and Comparison With Previous Literature

In this systematic review and meta-analysis, we critically examined the effects of nonpharmacological interventions, particularly regular PE, on treatment outcomes in hypertensive older adults. The study aimed to evaluate the efficacy of different PE modalities, including AT and RT as standalone interventions, as well as CT (aerobic and resistance), in reducing BP in this population. The findings suggest that CT is an effective option for reducing BP in older adults with hypertension; however, the evidence is characterized by very high between-study heterogeneity. Overall, all three exercise modalities were associated with reductions in BP, and CT may produce greater average reductions than AT or RT alone. Nevertheless, this apparent superiority should be interpreted with caution due to the limited number of direct comparisons and substantial variability across studies.

The observed reductions in SBP and DBP are generally consistent with prior evidence suggesting that exercise training can improve BP in patients with hypertension; however, effects across individual RCTs remain heterogeneous. One of the key findings is that AT significantly reduces both SBP and DBP. AT has been widely studied and is known to lower BP through mechanisms such as reduced systemic vascular resistance, increased nitric oxide bioavailability, and improved endothelial function, among others [12,18,48-50]. These findings are supported by previous meta-analyses reporting the effectiveness of AT in BP management among hypertensive patients, including those with CVD risk factors [11,51-55]. Similarly, consistent with our findings on RT, previous studies have shown that RT lowers BP by enhancing muscle strength, improving arterial compliance, and increasing overall physical activity levels [56-58]. Some evidence also suggests that RT may upregulate endothelial nitric oxide synthase (eNOS) expression and enhance nitric oxide bioavailability, thereby promoting endothelium-dependent vasodilation and contributing to BP reduction through improved vascular tone regulation. The antihypertensive effects of RT have also been supported by multiple prior meta-analyses [15,59-61].

However, the main findings of the present study confirm the effectiveness of CT for BP management compared with traditional exercise interventions. These findings provide additional insights beyond conventional exercise approaches and highlight the importance of nonpharmacological strategies in managing BP in older adults with hypertension. The results of a previous large meta-analysis, which included RCTs investigating endurance training, dynamic RT, CT (resistance and endurance), and isometric resistance training on resting BP in adults, demonstrated significant reductions in SBP across all exercise modalities: isometric resistance training (-10.9 mm Hg; 95% CI -14.5 to -7.4), endurance training (-3.5 mm Hg; 95% CI -4.6 to -2.3), dynamic RT (-1.8 mmHg; 95% CI -3.7 to -0.01), and CT (-1.4 mm Hg; 95% CI -4.2 to 1.5). Significant reductions in DBP were also observed following isometric RT (-6.2 mmHg; 95% CI -10.3 to -2.0), dynamic RT (-3.2 mmHg; 95% CI -4.5 to -2.0), endurance training (-2.5 mmHg; 95% CI -3.2 to -1.7), and CT (-2.2 mmHg; 95% CI -3.9 to -0.48) [11]. Although these findings are broadly consistent with our results, that study did not specifically focus on patients with clinically diagnosed hypertension; rather, it included trials assessing the effects of exercise interventions in general adult populations. In contrast, the present review focuses specifically on hypertensive older adults. Previous studies have nevertheless consistently demonstrated the beneficial effects of exercise interventions on BP management in hypertensive patients [50,62,63].

Notably, the limited number of studies directly comparing the effects of CT with AT or RT alone indicates that this remains an important area for further research. Our review addresses this gap by showing that CT, which combines AT and RT, induces complementary physiological adaptations that may lead to greater reductions in BP. The enhanced effect of CT is attributed to mechanisms associated with both exercise modalities. For instance, AT improves the efficiency of the cardiovascular and respiratory systems and enhances cardiovascular endurance, thereby contributing to BP reduction. RT, on the other hand, increases muscle strength and endurance and may improve arterial compliance by reducing peripheral vascular resistance. Together, these complementary adaptations may produce a synergistic effect, resulting in more pronounced improvements in BP regulation.

Clinical Implications

These findings have important implications for clinical practice and inform public health strategies for managing hypertension in older adults. They support the integration of structured exercise training, particularly appropriately prescribed aerobic, resistance, and combined programs, into routine hypertension care pathways and broader population-level strategies for this age group. Healthcare providers may consider prescribing CT as part of a comprehensive nonpharmacological management plan for older adults with hypertension. When implemented effectively, these programs not only reduce BP but also improve overall physical fitness, functional capacity, and quality of life. In addition, public health campaigns that promote physical activity can help increase participation in exercise programs, with particular emphasis on CT among older adults. Such initiatives should highlight the importance of regular physical activity in this population and provide the necessary resources and support to enable safe and sustained engagement.

Strengths, Limitations, and Future Directions

A key strength of this study is its rigorous methodological design, characterized by a comprehensive and systematically applied search strategy, clearly defined and consistently enforced inclusion criteria, and a structured risk‑of‑bias assessment for all included trials. A significant number of high-quality RCTs also add to the robustness and generalizability of the findings. Most importantly, a meta-analytic approach was used to aggregate data from multiple studies, thus providing a precise predicted effect of the assessed exercise modalities on BP.

However, several important limitations should be acknowledged. First, several pooled analyses showed very high between-study heterogeneity (I² often > 90%), arising from differences in participant characteristics, exercise protocols, follow-up durations, and outcome measures. This heterogeneity reduces the precision and generalizability of the pooled effect estimates. Although sensitivity analyses were performed, substantial residual heterogeneity likely remains. Second, participant numbers were unevenly distributed across exercise modalities, and some comparisons were based on a small number of trials and participants, limiting statistical power and the stability of subgroup analyses. Third, concomitant antihypertensive medication use was common but inconsistently reported across studies; therefore, medication effects may have confounded or influenced the observed BP changes. Fourth, the inclusion criteria were restricted to RCTs published as full-text articles in English-language peer-reviewed journals. As a result, potentially relevant non-English or unpublished studies may have been missed, introducing potential language and publication bias and possibly overestimating the true effects. Finally, variability in exercise duration and training protocols across studies limits the ability to define an optimal “dose” of AT, RT, or CT for older adults with hypertension. 

Because antihypertensive medication use was not consistently reported across studies, drug effects may have confounded or modified the observed BP changes attributed to exercise interventions.

Future research should explore the broader effects of CT on other clinically relevant outcomes in older adults with hypertension, such as lipid profiles, glycemic control, quality of life (including mental health outcomes), and cognitive function. Such evidence would further support the role of structured exercise as a comprehensive nonpharmacological intervention for hypertension management in older adults. Additionally, future meta-analyses incorporating longitudinal studies with longer follow-up periods are warranted to better assess the long-term efficacy of CT in BP control and to evaluate its broader cardiovascular benefits while improving standardization of intervention protocols and outcome measures.

## Conclusions

In summary, this systematic review and meta-analysis provide comprehensive evidence that CT interventions are more effective than other exercise modalities at lowering BP in older adults with hypertension. The findings indicate that, consistent with AT, which is widely recognized for its BP-lowering effects, RT also produces significant reductions in BP, particularly when compared with no exercise. A key novel finding is that the combination of AT and RT confers the greatest antihypertensive benefit in older adults with hypertension. These findings highlight the importance of incorporating CT protocols into both clinical practice and public health strategies to improve cardiovascular health and overall well-being in aging populations diagnosed with or receiving treatment for hypertension. Future research should further investigate and validate additional benefits of CT to optimize exercise interventions to improve health outcomes in older adults with hypertension.

## Appendices

Supplementary material 1 

**Table 3:** PRISMA checklist table BP: blood pressure, CT: combined training, AT: aerobic training, RT: resistance training, SBP: systolic blood pressure, DBP: diastolic blood pressure, PRISMA: Preferred Reporting Items for Systematic Reviews and Meta-Analyses, RCT: randomized controlled trial, RCTs: randomized controlled trials, N/A: not applicable.

Section and Topic	Item #	Checklist item	Location where the item is reported
TITLE			
Title	1	Identify the report as a systematic review	Cover page
ABSTRACT			
Abstract	2	See the PRISMA 2020 for Abstracts checklist	Abstract page
INTRODUCTION			
Rationale	3	Describe the rationale for the review in the context of existing knowledge	Introduction
Objectives	4	State clearly and explicitly the objective(s) or research question(s) addressed by the review	Introduction
METHODS			
Eligibility criteria	5	Describe the review’s inclusion and exclusion criteria and explain the approach used to categorize studies for the evidence syntheses	Methodology
Information sources	6	List every database, registry, website, organization, reference list, and any other source that was searched or consulted to identify eligible studies, and report the last search or consultation date for each source	Methodology
Search strategy	7	Provide the complete search strategies used for each database, registry, and website, clearly detailing all search terms, combinations, and any applied filters or limits	Methodology
Selection process	8	The predefined inclusion criteria were applied to all retrieved records, which were screened in two stages, title/abstract and then full text, by two reviewers working independently. Any disagreements regarding study eligibility were resolved through discussion, with arbitration by a third reviewer when needed, and no automated tools were used at any stage of the selection process	Methodology
Data collection process	9	Describe the procedures used for data extraction from the included reports, indicating the number of reviewers involved per study, whether they extracted data independently, any steps taken to request or verify information with study authors, and, if relevant, how any automated tools supported this process	Methodology
Data items	10a	List and briefly define every outcome for which data were extracted, and state whether all results corresponding to each outcome domain (across measures, time points, and analyses) were included for each study. If only a subset was used, clearly describe the decision rules or criteria applied to select which specific results were incorporated into the review	Methodology
	10b	List and clearly define all additional variables for which data were extracted (such as participant characteristics, intervention details, study setting, or funding and sponsorship). Also report any assumptions applied when information was missing, ambiguous, or incompletely reported, explaining how these assumptions were used to code or interpret the data	Methodology
Study risk of bias assessment	11	Risk of bias for the included studies was evaluated using a standardized tool (e.g., RoB 2), with each study assessed independently by two reviewers. Any discrepancies in judgments were resolved through discussion or, when necessary, by involving a third reviewer, and no automated methods were used in this appraisal	Methodology
Effect measures	12	State, for each outcome, which effect measure was applied (e.g., risk ratio, odds ratio, mean difference, or standardized mean difference) when conducting the synthesis and when reporting the results	Methodology
Synthesis methods	13a	Explain how you determined which studies contributed to each synthesis, for example, by summarizing and tabulating key intervention and comparator characteristics, then matching these against the predefined synthesis groups described in the eligibility criteria (item #5)	Methodology
	13b	Describe how data were readied for analysis and presentation, including the methods used to address missing summary statistics (e.g., imputation or calculation from available information) and any transformations or unit conversions applied before synthesis or reporting	Methodology
	13c	Describe how the findings from individual studies and from the overall syntheses were organized and presented, including any tabular formats or graphical displays (such as forest plots, summary tables, or other visual figures) used to illustrate the results	Methodology
	13d	Explain the statistical methods used to combine study results and justify the chosen approach. If a meta-analysis was conducted, specify the analytical model(s) applied (e.g., fixed‑effect or random‑effects), how statistical heterogeneity was assessed and quantified (e.g., using the I² statistic or related tests), and identify the software used to carry out these analyses	Methodology
	13e	Describe the approaches used to investigate potential sources of variation in effect estimates across studies, such as planned subgroup analyses, sensitivity analyses, or meta‑regression, and indicate how these analyses were specified and interpreted	Methodology
	13f	Describe any sensitivity analyses performed to test the stability of the pooled estimates, including which studies, assumptions, or analytic choices were varied or excluded, and how these changes affected the overall findings	Methodology
Reporting bias assessment	14	Explain how you evaluated the potential for bias arising from missing or selectively reported results within each synthesis, such as by examining small‑study effects (e.g., with funnel plots or statistical tests) or comparing registered protocols and trial reports with published outcomes, and how these assessments informed your judgments about reporting bias	Methodology
Certainty assessment	15	Describe the approach used to rate the overall certainty (or confidence) in the evidence for each outcome, such as applying a structured framework (e.g., GRADE), including which domains were considered (e.g., risk of bias, inconsistency, indirectness, imprecision, and publication bias) and how judgments across these domains were combined to arrive at a final certainty rating	Methodology
RESULTS			
Study selection	16a	Report the outcome of the search and study selection process by detailing how many records were initially retrieved, how many were excluded at each screening stage (e.g., duplicates, title/abstract, full text), and how many studies were ultimately included in the review, preferably summarizing this process in a PRISMA-style flow diagram	Results section
	16b	List the studies that appeared eligible based on initial screening but were ultimately excluded, and for each, provide a brief justification for exclusion (e.g., wrong population, intervention, comparator, outcome, or study design)	N/A
Study characteristics	17	Each citation includes a study and presents its characteristics	Results section
Risk of bias in studies	18	Present assessments of risk of bias for each included study	Results section
Results of individual studies	19	For each outcome, present, for every included study: (a) the summary statistics by group (where relevant) and (b) the corresponding effect estimate together with a measure of precision (such as a confidence or credible interval), preferably using structured tables or graphical displays (e.g., forest plots) to organize these data	Results section
Results of syntheses	20a	For each synthesis, provide a concise overview of the key study features (such as populations, interventions, comparators, settings, and follow‑up durations) and summarize the overall risk‑of‑bias judgments for the contributing trials, noting any common or serious methodological limitations that could influence the pooled results	Results section
	20b	Report the findings of every statistical synthesis performed. For each meta-analysis, provide the pooled effect estimate with its measure of precision (such as a confidence or credible interval) and the corresponding heterogeneity statistics (e.g., I², heterogeneity P value), and, when groups are compared, clearly indicate which group the effect favors and the direction of the difference	Results section
	20c	Report the findings from all analyses undertaken to explore potential sources of heterogeneity (e.g., subgroup analyses, sensitivity analyses, or meta‑regression), describing which factors were examined and how they influenced the effect estimates and between‑study variability	Results section
	20d	Report the outcomes of every sensitivity analysis performed, indicating which studies, assumptions, or analytic decisions were varied or excluded, and describe how these changes affected the pooled effect estimates and your overall conclusions about the results’ robustness	Results section
Reporting biases	21	Report, for each synthesis, your judgments about the risk of bias arising from missing or selectively reported results, describing any evidence of reporting bias (e.g., funnel plot asymmetry or discrepancies between planned and published outcomes) and how this influenced your confidence in the findings	Results section
Certainty of evidence	22	Summarize the certainty (or confidence) of the evidence for each outcome using a structured framework (such as GRADE), stating the final rating (high, moderate, low, or very low) and briefly outlining the main reasons for any downgrading or upgrading decisions	Results section
DISCUSSION			
Discussion	23a	Provide an overarching interpretation of the findings, placing them in the context of prior research and the existing evidence base, and highlighting how your results align with, contrast, or extend previous studies	Summary of findings (discussion)
	23b	Describe the main weaknesses and gaps in the underlying evidence included in this review, such as methodological flaws, inconsistency or imprecision of results, indirectness of populations or interventions, and any concerns about reporting or publication bias	Limitations (Discussion)
	23c	Outline the key constraints and potential sources of bias arising from the review process itself, such as restrictions in the search strategy, eligibility criteria, data extraction or risk‑of‑bias procedures, synthesis methods, or deviations from the registered protocol, and explain how these may have influenced the findings	Limitations (Discussion)
	23d	Discuss how these findings should inform clinical decision-making and day-to-day care, highlight their potential impact on health policy or guideline development, and identify key priorities or gaps that future research should address	Limitations (Discussion)
OTHER INFORMATION			
Registration and protocol	24a	Report the review’s registration information by naming the registry and providing the registration number; if no registration was completed, explicitly state that the review was not prospectively registered	N/A
	24b	State where the review protocol is available (e.g., a registry, institutional repository, or journal publication), including any relevant identifiers, or clearly indicate that no formal protocol was developed for this review	N/A
	24c	Describe any changes made to the methods or plans specified at registration or in the original protocol, explaining what was modified, when the change occurred, and the rationale for doing so	N/A
Support	25	Describe all financial and nonfinancial support received for conducting the review (such as grants, institutional resources, or in‑kind contributions) and clarify what, if any, involvement funders or sponsors had in the design, conduct, analysis, or reporting of the review	Financial Disclosure
Competing interests	26	Disclose any potential conflicts of interest among the review authors, including financial, professional, or personal relationships that could be perceived as influencing the conduct or reporting of the review	Conflict of Interest
Availability of data, code, and other materials	27	Indicate which review materials are publicly accessible, such as template data collection forms, extracted study data, datasets used in the analyses, analytic code, and any additional supporting documents, and specify where each can be found (e.g., in an online repository or as supplementary files)	Supplementary files

**Table 4:** Data sheet 1: full search strategy for the electronic databases queried (PubMed MEDLINE)

PubMed (MEDLINE) Platform: National Library of Medicine N/B: PubMed was searched with appropriate Medical Subject Headings (MeSH), with filters set for Humans: ("hypertension"[MeSH Terms] OR "hypertension"[MeSH Major Topic]) AND ("older adults"[Title/Abstract] OR "elderly"[Title/Abstract]) AND ("exercise"[MeSH Major Topic] OR "exercise"[Title/Abstract] OR "exercise"[MeSH Terms] OR (("mixed"[All Fields] OR "mixes"[All Fields] OR "mixing"[All Fields] OR "mixings"[All Fields]) AND "exercise"[MeSH Terms]) OR "resistance training"[MeSH Terms] OR "resistance training"[MeSH Terms] OR (("combinable"[All Fields] OR "combined"[All Fields] OR "combination"[All Fields] OR "combinational"[All Fields] OR "combinations"[All Fields] OR "combinative"[All Fields] OR "combine"[All Fields] OR "combined"[All Fields] OR "combines"[All Fields] OR "combining"[All Fields]) AND "exercise"[MeSH Terms])) AND "randomized controlled trial"[Publication Type]

**Table 5:** GRADE checklist for evidence quality (A) Random sequence generation (selection bias). (B) Allocation concealment (selection bias). (C) Blinding of participants and practitioners (performance bias). (D) Blinding of outcome assessment (detection bias). (E) Objective outcomes used. (F) More than 80% of participants were included in the analysis (reporting bias). (G) Consistent reporting of outcome of interest (selective reporting). (H) Trial completed as scheduled (no early stopping). (I) Random sequence generation used (selection bias, duplicate check). Scores: 1 = yes, 0 = uncertain, -1 = no, NA1 = not evaluated, and NA2 = not applicable. GRADE: Grading of Recommendations Assessment Development and Evaluation.

Study	Study limitations (risk of bias)									Inconsistency							Indirect evidence						Imprecision					Publication bias					GRADE evidence quality
Scale score	A	B	C	D	E	F	G	H	I		A	B	C	D	E	A		B	C	D	E	A		B	C	D	A	B	C	D	E	F	
Bavaresco Gambassi et al. (2024) [31]	1	1	0	1	1	1	1	1	1		1	NA1	1	Low	1	1		1	1	1	1	1		NA2	0	1	1	NA2	NA2	NA2	NA2	NA2	High
Bavaresco Gambassi et al. (2023) [33]	1	1	0	1	1	1	1	1	1		1	NA1	1	Low	1	1		1	1	1	1	1		NA2	0	1	1	NA2	NA2	NA2	NA2	NA2	High
Hou et al. (2023) [32]	1	1	0	1	1	1	1	1	1		1	NA1	1	High	0	1		0	1	1	1	1		NA2	0	1	1	NA2	NA2	NA2	NA2	NA2	Moderate
Abrahin et al. (2022) [34]	1	1	1	1	1	1	1	1	1		1	NA1	1	Low	1	1		1	1	1	1	1		NA2	0	1	1	NA2	NA2	NA2	NA2	NA2	High
Abrahin et al. (2022) [35]	1	1	0	1	1	1	1	1	1		1	NA1	1	Low	1	1		1	1	1	1	1		NA2	0	1	1	NA2	NA2	NA2	NA2	NA2	High
Gargallo et al. (2022) [36]	1	1	0	1	1	1	1	1	1		1	NA1	1	Low	1	1		1	1	1	1	1		NA2	0	1	1	NA2	NA2	NA2	NA2	NA2	High
Sardeli et al. (2022) [37]	1	1	0	1	1	1	1	1	1		1	NA1	1	High	0	1		1	1	1	1	1		NA2	0	1	1	NA2	NA2	NA2	NA2	NA2	High
Caminiti et al. (2021) [30]	1	1	0	1	1	1	1	1	1		1	NA1	1	Moderate	1	1		1	1	1	1	1		NA2	0	1	1	NA2	NA2	NA2	NA2	NA2	High
Kaholokula et al. (2021) [38]	1	1	0	1	1	1	1	1	1		1	NA1	1	Moderate	1	1		1	1	1	1	1		NA2	0	1	1	NA2	NA2	NA2	NA2	NA2	High
Lopes et al. (2021) [39]	1	1	1	1	1	1	1	1	1			NA1	1	Moderate		1		1	1	1	1	1		NA2	0	1	1	NA2	NA2	NA2	NA2	NA2	High
Iellamo et al. (2021) [40]	1	1	0	1	1	1	1	1	1		1	NA1	1	High	0	1		1	1	1	0	1		NA2	0	1	1	NA2	NA2	NA2	NA2	NA2	Moderate
Da Silva et al. (2020) [41]	1	1	0	1	1	1	1	1	1		1	NA1	1	Low	1	1		1	1	1	1	1		NA2	0	1	1	NA2	NA2	NA2	NA2	NA2	High
Schimitt et al. (2020) [42]	1	1	0	1	1	1	1	1	1		1	NA1	1	Moderate	1	1		1	1	1	1	1		NA2	0	1	1	NA2	NA2	NA2	NA2	NA2	High
Sócrates et al. (2020) [43]	1	1	1	1	1	1	1	1	1		1	NA1	1	High	0	1		1	1	1	0	1		NA2	0	1	1	NA2	NA2	NA2	NA2	NA2	Moderate
Schroeder et al. (2019) [44]	1	1	0	1	1	1	1	1	1		1	NA1	1	Low	1	1		1	1	1	1	1		NA2	0	1	1	NA2	NA2	NA2	NA2	NA2	High
de Oliveira et al. (2019) [45]	1	1	1	1	1	1	1	1	1		1	NA1	1	Low	1	1		1	1	1	1	1		NA2	0	1	1	NA2	NA2	NA2	NA2	NA2	High
Ruangthai et al. (2019) [46]	1	1	1	1	1	1	1	1	1		1	NA1	1	Low	1	1		1	1	1	1	1		NA2	0	1	1	NA2	NA2	NA2	NA2	NA2	High
Chan et al. (2018) [47]	1	1	0	1	1	1	1	1	1		1	NA1	1	Moderate	1	1		1	1	1	1	1		NA2	0	1	1	NA2	NA2	NA2	NA2	NA2	High
Dantas et al. (2016) [48]	1	1	0	1	1	1	1	1	1		1	NA1	1	Moderate	1	1		1	1	1	1	1		NA2	0	1	1	NA2	NA2	NA2	NA2	NA2	High
	A. Was the allocation sequence generated using an appropriate random method, indicating a low risk of selection bias? B. Was the concealment of group assignment adequately maintained, so that upcoming allocations could not be predicted (minimizing selection bias)? C. Were participants and treating personnel unaware of the intervention assignments, thereby reducing performance bias? D. Were those who measured or evaluated outcomes blinded to group allocation, limiting the potential for detection bias? E. Were outcomes assessed using clearly defined, objective measures or standardized instruments? F. Were at least 80% of the participants originally randomized included in the primary analysis, suggesting a low risk of attrition or reporting bias? G. Was the outcome of interest reported completely and consistently across all study arms, with no indication of selective outcome reporting? H. Did the trial proceed to completion as planned, without being terminated prematurely?									A. Does the pooled point estimate show limited dispersion across studies (i.e., is the central effect size relatively stable)? B. To what extent do the confidence intervals for the individual study estimates overlap? All: For at least one effect estimate, the confidence intervals of every included study intersect. Some: Confidence intervals overlap partially, but not all studies share overlap at any single effect estimate. None: At least one study behaves as an outlier, such that the confidence intervals of several studies do not overlap for most effect estimates. C. Is the direction of the effect generally consistent across studies (i.e., do most studies favor the same side of the null)? D. What is the degree of heterogeneity as indicated by the I² statistic? Low: I² < 40% Moderate: I² = 40%-60% High: I² > 60% E. Is the statistical test for heterogeneity not significant (p > 0.10), suggesting that observed variability might be compatible with chance?							A. Are the populations enrolled in the included studies sufficiently similar to the target population for whom clinical or policy decisions will be made? B. Are the interventions evaluated in the included studies relevant and feasible in the actual decision‑making setting? C. Are the outcomes assessed directly meaningful to patients, rather than primarily surrogate or intermediate endpoints? D. Was the length of follow‑up adequate for the outcomes of interest to reasonably occur and be detected? E. Are the main conclusions drawn from head‑to‑head (direct) comparisons between interventions, rather than relying predominantly on indirect evidence?						Here is a rephrased version for the Imprecision domain, with the same meaning but noticeably different wording: A. Is the confidence interval around the pooled effect estimate from the meta‑analysis sufficiently narrow and compatible with either benefit or harm (i.e., not spanning both clearly)? B. What is the approximate median sample size of the contributing studies (high: >300 participants; intermediate: 100-300 participants; low: <100 participants)? C. How many studies contribute data to the pooled estimate (large: >10 studies; moderate: 5-10 studies; small: <5 studies)? D. Is the outcome relatively frequent (e.g., occurring in more than 1 in 100 participants), rather than being a very rare event?					A. Was the literature search wide‑ranging and comprehensive in scope? B. Was gray literature (e.g., theses, trial registries, conference abstracts) actively searched for and considered? C. Were studies not excluded on the basis of publication language (i.e., no language restrictions applied during study selection)? D. Is there no clear indication that industry funding or sponsorship could systematically influence the body of evidence included in the review? E. Is there visual evidence of asymmetry in funnel plots or similar tools used to explore small‑study effects? F. Are there no important discrepancies between results reported in published sources and those available from unpublished data for the included studies?					
